# Study on the geometry characteristics of soil primary mineral particles under cryogenic action

**DOI:** 10.1038/s41598-022-21023-8

**Published:** 2022-10-06

**Authors:** Jinbang Zhai, ShengRong Zhang, Ze Zhang, Andrey Melnikov, Hang Li

**Affiliations:** 1 School of Transportation/Institute of Cold Regions Science and Engineering, Northeast Forestry University, Harbin, 150040 People’s Republic of China; 2 School of Civil Engineering/Northeast-China Observatory and Research-Station of Permafrost Geo-Environment of the Ministry of Education, Northeast Forestry University, Harbin, 150040 People’s Republic of China; 3 Melnikov Permafrost Institute, Siberian branch, Russian Academy of Science, Yakutsk, 677010 Russian Federation

**Keywords:** Cryospheric science, Geology

## Abstract

Repeated freeze–thaw causes the fragmentation and aggregation of soil particles, which affect particle shape (aspect ratio, roundness, etc.), and this process is a cryogenic weathering process. Changes in soil particle morphology record information about freeze–thaw processes and have the unique characteristics of freeze–thaw traces. To prove this conjecture, four soil specimens were selected in the experiment, and each specimen was studied after 0, 5, 10, 50 and 100 freeze–thaw cycles. The test results show that: Freeze–thaw will change the aspect ratio of particles, and the aspect ratio of particles is mainly distributed between 1 and 4. The particles with aspect ratio of 1.26 are stable and not easy to fragment, and the particles with aspect ratio more than 4 are easy to fragment. The freeze–thaw effect leads to changes in particle roundness, with different manners of change for the four specimens, but all undergo repeated freeze–thaw fragmenting and rounding process. Repeated freezing and thawing can not only lead to fragmentation particle edges and increased particle roundness, but also to fragmentation large-size particles and reduced particle roundness. Compared with the roundness before freeze–thaw and after 100 cycles of freeze–thaw, the coarse sand grains increased the most in roundness, indicating that the large grain size grains showed the most rounding. This study helps to understand the geometric characteristics of soil primary mineral particles under the action of cryogenic environments, and also helps to discern whether the particles have experienced the action of cryogenic environments, which is important for the study of cryogenic soil in cold environments.

## Introduction

The phase transition of water, ice crystal growth and water migration during the freezing and thawing process will affect the soil structure and generate forces between soil particles. In confined volume, the expansion pressure can be close to 1.4 × 10^4^ kPa during ice formation^1^. The repeated freezing and thawing process will fragment the soil particles^2^, and then change the soil particle size. The freeze–thaw test shows that the freeze–thaw process will lead to the fragmentation of coarse mineral particles and the aggregation of fine particles^3^, and the fragmentation of coarse particles and the aggregation of fine particles are synchronous^4^.

The freezing and thawing process will not only change the size of soil particles and affect the distribution of grain size, but also change the particle’s shape. The particle’s shape and surface texture provide vital information regarding the origin, transport and deposition history^5–11^. The initial shape of particles depends on rock parent material and weathering, and the final shape depends on the transport medium and transport mode. When the transport is the fluvial medium, the average particle size, skewness (Skewness is a reflection of sediment grain size), lithology, shape and roundness are a function of distance. The rate of rounding is directly proportional to some power of the distance transported^12^. Abundant abrasion features like V-shaped pits, sharp edges, linear and curved grooves usually give evidence of transport in a fluvial medium^13,14^. In an aeolian environment, due to particle size selective transport and saltation abrasion, most of particle shape contain well-sorted, subrounded grains^15–18^. In addition, the mineral components are mainly quartz, feldspar and calcite. Aeolian quartz features include meandering ridges, upturned plates, bulbous edges, and adhering particles on quartz^14^.

However, there are few studies on the characteristics of particle shape during the freeze–thaw process (cryogenic weathering). Previous studies have shown that the fragmentation and aggregation of soil particles causes the phenomenon of silt-level enrichment^3,19–22^, which is unique physical weathering phenomenon of cryogenic soil. The degree of siltylation of cryogenic soil is directly associated to factors such as freeze–thaw intensity and duration, especially the long-term fluctuating climate with alternating cold and warm will promote its development process^4,23,24^.

The shape and surface structure of the grains, as found through the above literature, provide important information about the origin, transport and deposition history. However, there are fewer studies on the morphological characteristics of particles during freeze–thaw (cryogenic weathering). In order to search for the unique morphological characteristics of particles under cryogenic weathering, we performed freeze–thaw tests on four types of soil samples. It is hoped that this study will find the unique morphological characteristics of soil primary mineral particles under the action of cryogenic environment.

## Materials and methods

### Test soil specimens

The test selected loess (L), coarse sand (CS), very fine sand (VFS) and fine sand (FS) as the test objects. The physical properties and distribution of grain size of the four soil specimens are shown in Table 1.

**Table 1:** Physical properties of four type soil samples and test methods.

Test method	Physical properties	Specimen (L)	Specimen (CS)	Specimen (VFS)	Specimen (FS)
Cutting ring method	Density (g/cm^3^)	2.04	2.02	2.06	2.08
Oven-drying method	Water content (%)	24.26	18.36	22.13	19.11
Casagrande method	Liquid limit W_L_ (%)	28.02	–	–	–
Thread-rolling method	Plastic limit W_P_ (%)	23.21	–	–	–
W_L_ − W_P_	Plastic index I_P_ (%)	4.81	–	–	–

### Test equipment

**Figure 1:**
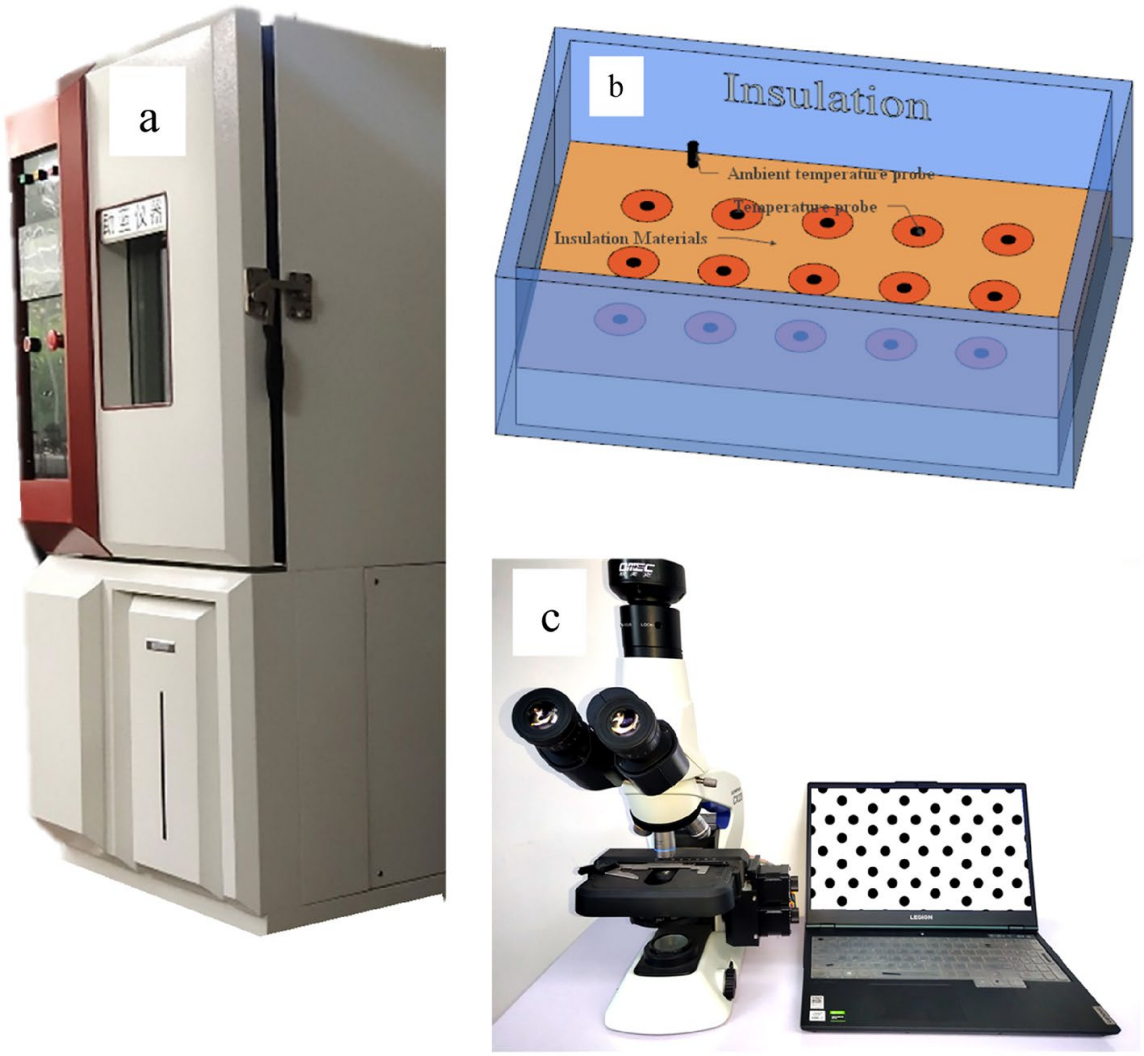
Test device: (**a**) Freeze–thaw test device; (**b**) Schematic diagram of freeze–thaw test for soil samples; (**c**) Particle Image Processor.

The freeze–thaw cycles test of the soil specimens was carried out in the freeze–thaw cycles test chamber (Fig. 1a). The model of the freeze–thaw cycle test chamber is ZLHS-250-LS. The soil specimen was saturated by the vacuum-pumping saturation method^25^, and the specimens were sealed with cling film. The temperature test probe in the specimen is used to determine whether the soil sample is completely frozen and thawed, and the specimen ambient temperature of the test is determined from the ambient temperature probe. And insulation material is used to ensure that the soil sample freezes in one direction (Fig. 1b).

**Figure 2:**
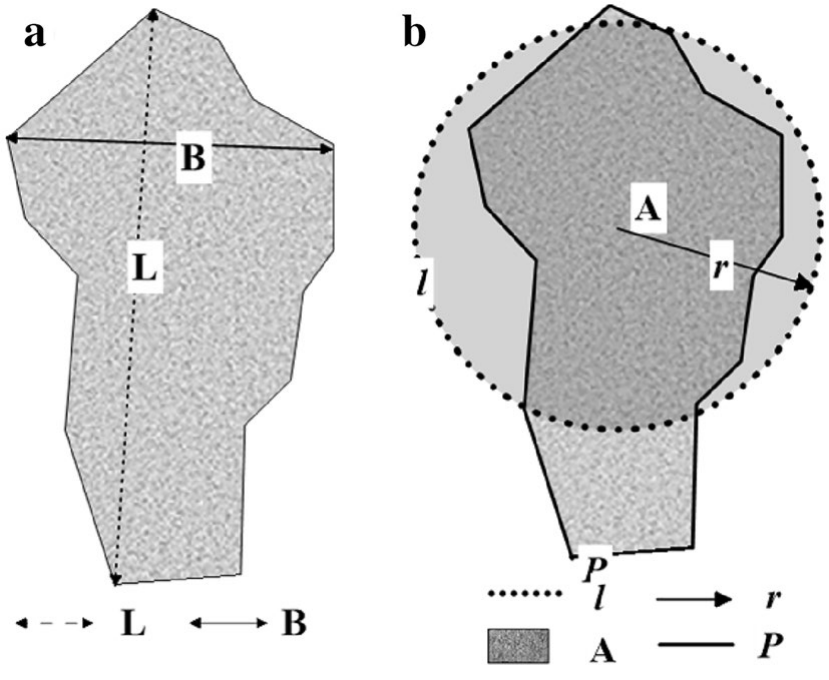
Schematic diagram of aspect ratio (**a**) and roundness (**b**).

In order to analyze the particle shape changes of soil specimens after freezing and thawing cycles, a particle image processor (Fig. 1c) was used to observe the soil specimens after freezing and thawing. The measurement range of particle image processor used in the test is 0.5 μm to 3000 μm; Repetition accuracy: 1%. The data processing method of the particle image processor: measurement background and adjustment, particle image conversion and transmission, particle image binarization, particle edge search, calculation of particle parameters, analysis statistics and analysis results output. The computer automatically identifies the edges of the particles based on the received binarization particle image signal and then automatically calculates the particle size, aspect ratio and roundness of each particle. Typically, an image (i.e. a field of view of the imager) contains from a few to hundreds of particles. The imager automatically calculates and counts all the particles in the field of view to produce a report. When not enough particles are measured, the microscope can be adjusted to move to the next field of view and continue testing and counting. The particle image processor can output data such as aspect ratio (Fig. 2a), roundness (Fig. 2b), specific surface area, and distribution of grain size.

### Test program

The four types of soil specimens were air-dried naturally, crushed, and sieved (2 mm), and the soil specimens were used for freeze–thaw cycles tests. The freeze–thaw test was set at − 20 °C for freezing and + 20 °C for thawing. We conducted preliminary experiments on specimens to determine the maximum freeze–thaw time. The time required for the soil specimens to be completely frozen and thawed is 4 h. Thus, the freeze–thaw cycles period is 8 h. The use of a ring knife (diameter 61. 8 mm, 20 mm high) to minimize the influence on the physical parameters of the specimens, so as to assure the unity of the specimens and the credibility and contrast of the test results. The prepared specimens were vacuum-saturated and sealed up and down with cling film to maintain the closed state of the system conditions. This experiment was planned to take specimens and tests after 0, 5, 10, 50, and 100 freeze–thaw cycles. According to the number of freeze–thaw cycles (0, 5, 10, 50, 100), a total of 6 sets of specimens were required for each type of soil.

After the specimens were ready, the freeze–thaw cycles test chamber was used to perform freeze–thaw cycles on the specimens. After reaching the number of freeze–thaw cycles, the specimens were taken out for testing, and the remaining specimens continued to freeze–thaw cycles tests until all the planned freeze–thaw cycles were completed. After the freeze–thaw cycle, the specimens were tested and analyzed by the particle image processor. The measured data such as aspect ratio and roundness were analyzed (Table 2).

**Table 2:** Definition and schematic diagram of particle aspect ratio and roundness.

Particle geometry characteristics	Definition	Formula	Schematic diagram	Physical meaning
Aspect ratio	The ratio of the longest diameter passing through the interior of the particle to the shortest diameter perpendicular to the long diameter	$$\begin{array}{c}\theta =\frac{L}{B}\end{array}$$ (1)	Fig. 2a	The aspect ratio can represent the elongation properties of the particles. The closer the value of the aspect ratio is to 1, the closer the particle is to a square or a circle. The larger the value of the aspect ratio, the more elongated the particle is
Roundness	The ratio of the perimeter of the equivalent area circle of the particle projection to the perimeter of the particle projected contour	$\mathrm{\varnothing }=\frac{l}{P}$ (2)^26^	Fig. 2b	Generally, the smaller the value, the longer the projected particle contour and the more the particle shape deviates from the circle

(*θ*: aspect ratio; $L$: longest diameter inside the particle; *B*: shortest diameter perpendicular to the longest diameter; $\varnothing$: roundness; *A*: projected area of particles ($A=\pi {r}^{2}$ (3)); *l*: perimeter of the equivalent area circle of the particle projection ($l=2\pi r$ (4)); *r*: equivalent circle radius of particles; *P*: perimeter of the particle projected contour; Substitute formula (3) and (4) into formula (2) to obtain the roundness formula: $\mathrm{\varnothing }=\frac{2\sqrt{\pi A}}{P}$).

## Results and analysis

### Analysis of particle aspect ratio

**Figure 3:**
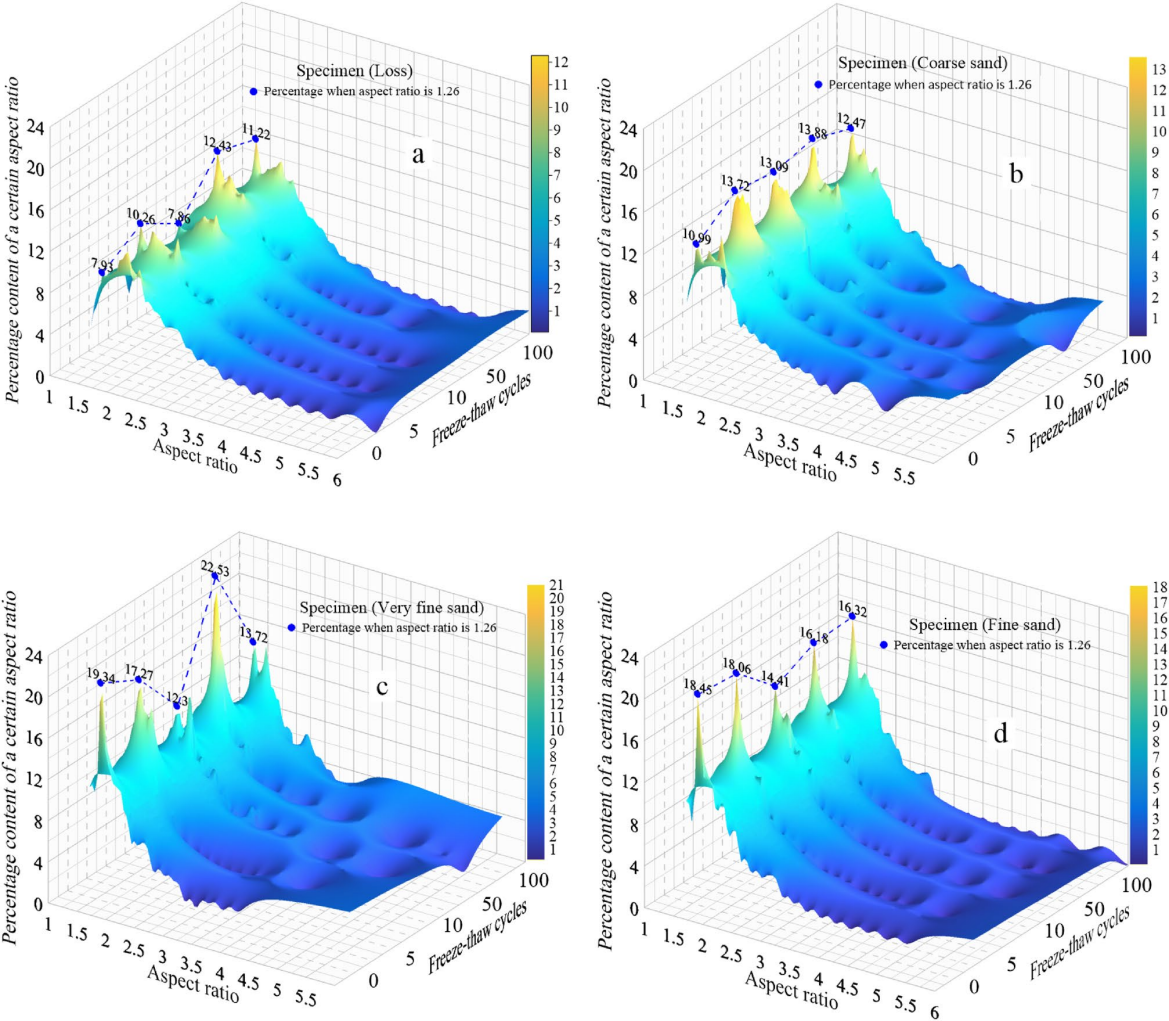
The percentage content change of a certain aspect ratio of four soil specimens after different freeze–thaw cycles. (**a** Specimen(L); **b** Specimen (CS); **c** Specimen (VFS); **d** Specimen (FS)).

Figure 3 shows the percentage content change of a certain aspect ratio following 0, 5, 10, 50, and 100 freeze–thaw cycles, with comparisons presented within each soil particle size. The figure shows that the aspect ratio of particles before and after freeze–thaw cycle is distributed between 1 and 6. Among four specimens, the value of aspect ratio between 1 and 4 exceeded 98%, indicating that the aspect ratio of the specimens were mainly distributed between 1 and 4, which meant that particles with an aspect ratio greater than 4 were prone to fragment. The peak value of four specimens was between 1 and 2 (e.g., the percentage of particles with an aspect ratio of 1.26 is 12.43 after the 50th freeze–thaw cycle for the specimen (loess)). The peaks of specimen (L) and specimen (CS) are gentle, while the peaks of specimen (VFS) and specimen (FS) are steep.

The dominant aspect ratio following higher amounts of freeze–thaw was 1.26, with over 12.43% of the four specimens showing an aspect ratio of 1.26 after 50 freeze–thaw cycles, and 11.22% after 100 freeze–thaw cycles, which indicates that the particles state is stable and not easily fragmented when the aspect ratio is 1.26. With the increase of freeze–thaw cycles, the percentage of particle aspect ratio increased or decreased, which indicated that the freeze–thaw cycles can change the particle’s aspect ratio. On the whole, the aspect ratio of particles shows a decreasing trend, that is, the proportion of particles with large aspect ratio decreases, and the proportion of particles with small aspect ratio increases, which indicates that the shape of particles is closer to circular or square with increasing freeze–thaw cycles.

**Figure 4:**
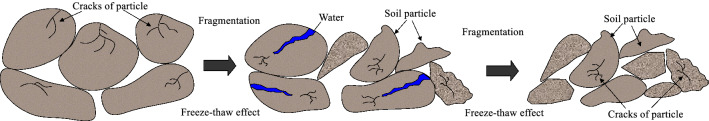
Conceptual diagram of the change in aspect ratio of large particle size due to freeze–thaw cycles.

In order to better represent the change of aspect ratio of particles after freezing and thawing, the particle fragmenting process is described in the form of conceptual diagram. As shown in Fig. 4. The change process of particle aspect ratio is as follows: under the effect of temperature and tension, cracks are formed on the surface of soil particles. The water in the soil enters into the crack, and the phase change of water in the crack causes the crack to further develop and fragment. The fragmentation of the particles eventually leads to a change in the aspect ratio of the particles. It should be pointed out that the aspect ratio of the larger particle diameter may be smaller, and vice versa.

**Figure 5:**
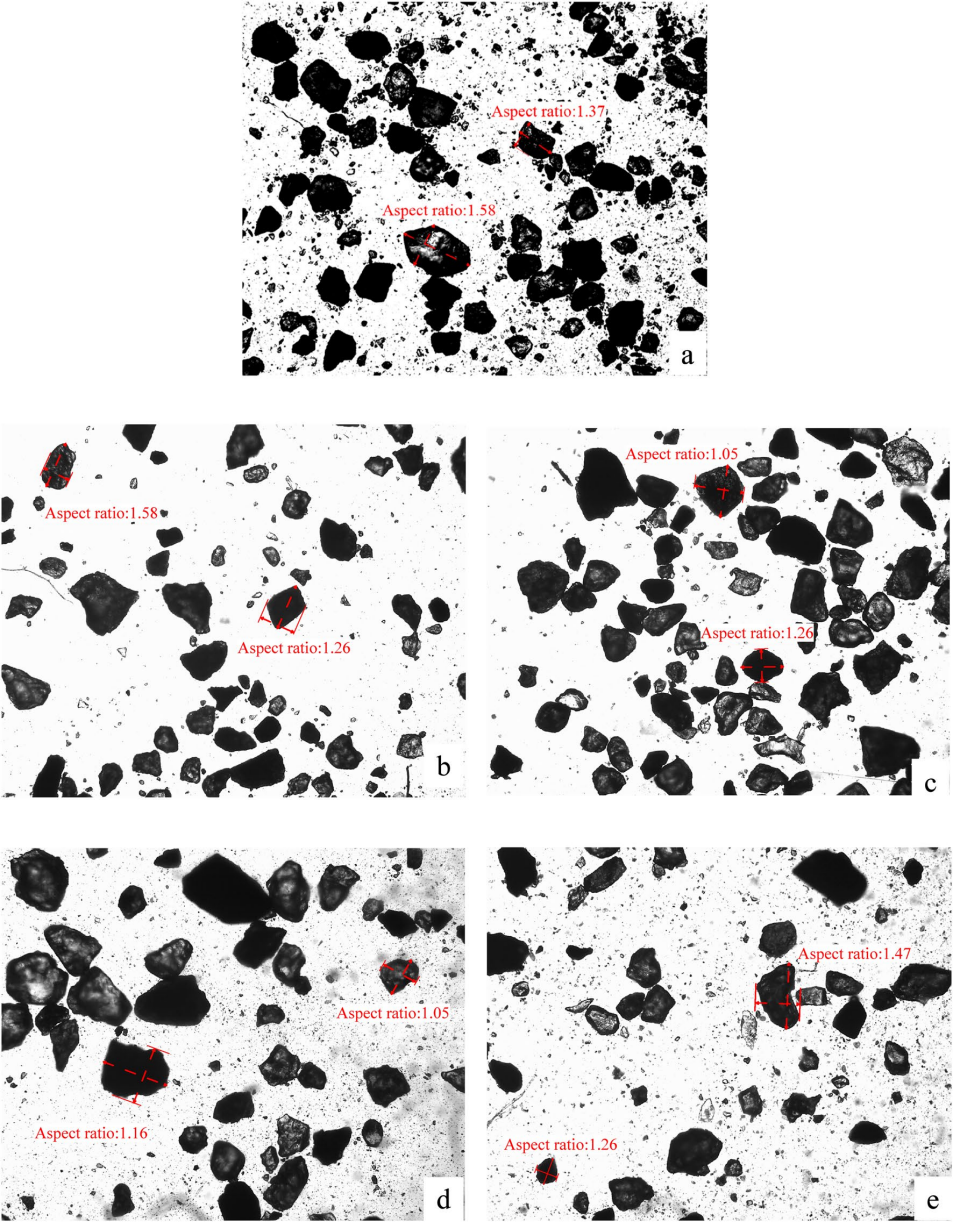
Particle size image of the specimen (CS) after freeze–thaw cycles. (**a** 0 freeze–thaw cycles; **b** 5 freeze–thaw cycles; **c** 10 freeze–thaw cycles; **d** 50 freeze–thaw cycles; **e** 100 freeze–thaw cycles).

The grain size of specimen (CS) after freeze–thaw cycles were used to analyze the change of particle aspect ratio. It can be seen from Fig. 5 that the aspect ratio of particles changed after freeze–thaw. The shape of particles is mainly polygonal, rectangular or elliptical, and the strip shape is very few, that is, the proportion of particles with larger aspect ratio is less, which indicates that particles with larger aspect ratio are easily fragmented. In addition, it was found that the proportion of particles with smaller aspect ratio was larger. This is consistent with the analysis results of specimen (CS) in Fig. 3.

### Analysis of particle roundness

**Figure 6:**
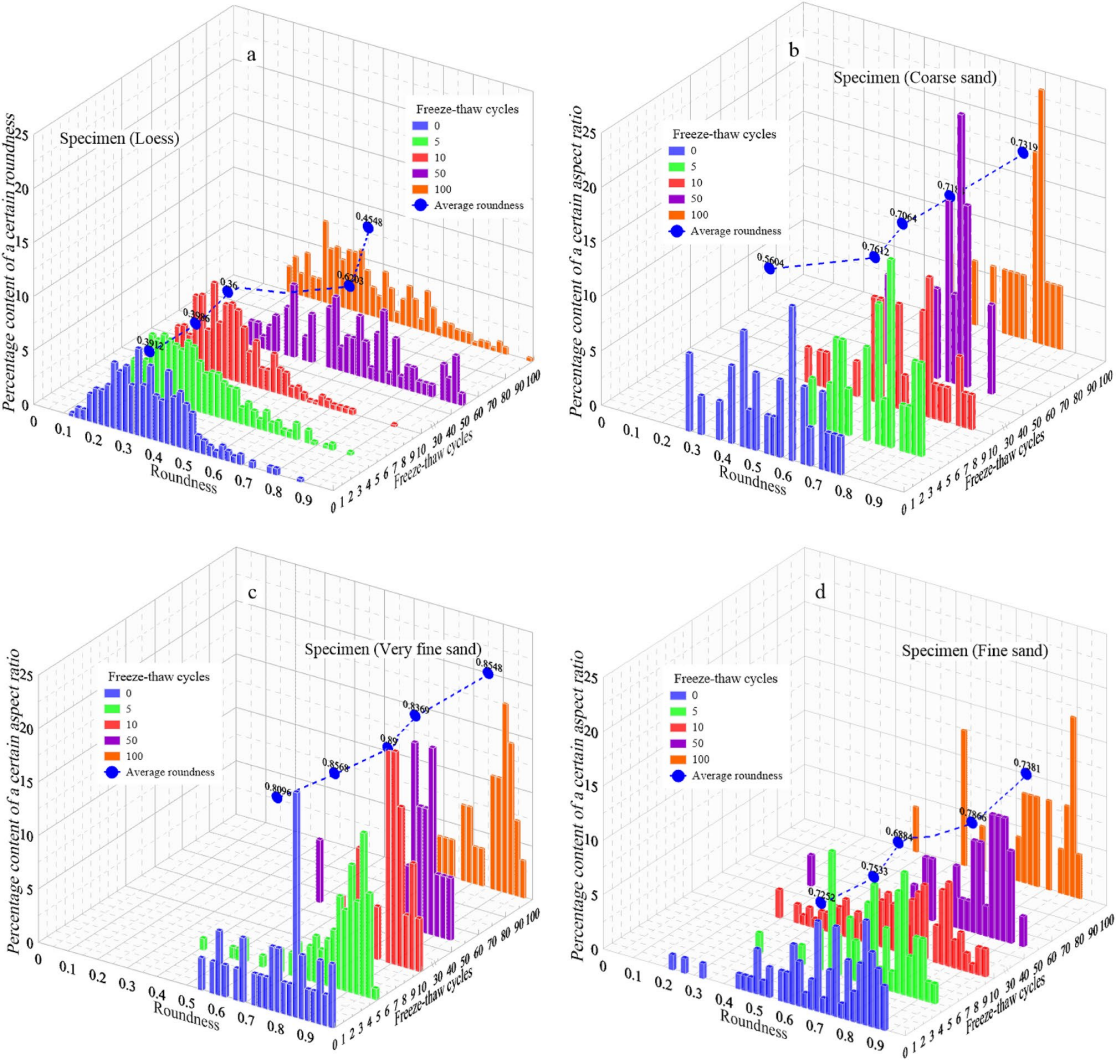
The percentage content change of a certain roundness of four soil specimens after different freeze–thaw cycles. (**a** Specimen (L); **b** Specimen (CS); **c** Specimen (VFS); **d** Specimen (FS)).

Figure 6 is the percentage change of particle roundness of four specimens after different freeze–thaw cycles. The blue dotted line in the figure is the percentage change curve of the average roundness of particles after freeze–thaw cycles. It can be seen from the figure that percentage content changed after freeze–thaw cycles, which shows that the particles roundness changed after freeze–thaw. Overall, with the increase of freeze–thaw cycles, the percentage content of small roundness value decreased, and the percentage content of large roundness value increased, which indicated that the particle shape was closer to a circle. This corresponds to an increase in aspect ratio for small values and a decrease in aspect ratio for large values. In addition, the roundness span of specimen (L) is the largest, followed by specimen (FS) and specimen (CS), and the smallest of specimen (VFS). However, on the whole, the roundness of specimen (VFS) is the largest and most concentrated.

Compared with the roundness before freeze–thaw and after 100 freeze–thaw cycles, the roundness of loess increased by 0.0636, that of coarse sand increased by 0.1715, that of very fine sand increased by 0.0452, and that of fine sand increased by 0.0129. It can be seen that the roundness of coarse sand increased the most, which indicates that the roundness of particles with large particle size increased the most.

**Figure 7:**
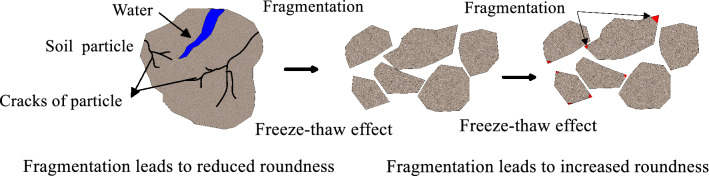
Conceptual diagram of the change in particles roundness due to freeze–thaw cycles.

Specifically, specimen (VFS) is taken as an example for analysis. From the roundness change diagram of specimen (VFS), It can be seen that the particle’s roundness increases as a whole after 5 freeze–thaw cycles, and the roundness of particles also is increased as a whole after the 10th freeze–thaw cycle. This shows that after 5 freeze–thaw cycles, the particle shape becomes rounder, after 10 freeze–thaw cycles, the particle shape becomes more rounder, which means that in the process of 0–10 freeze–thaw cycles, the freeze–thaw effect increases the particles roundness. However, after the 50th freeze–thaw cycle, compared with the particle roundness after the 10th freeze–thaw cycle, the particle roundness is decreased, which is due to the freeze–thaw effect leading to particle fragmentation and resulting in the decrease of particle roundness. After 100 freeze–thaw cycles, the particles roundness increases again and is larger than that after 10 freeze–thaw cycles, which indicates that freeze–thaw effect increases the particles roundness again. That is to say, in the process of 0–100 freeze–thaw cycles, specimen (VFS) experienced the process of first rounding (0–10 freeze–thaw cycles), then fragmenting (10–50 freeze–thaw cycles) and finally rounding (50–100 freeze–thaw cycles). That is, as illustrated in the concept diagram in Fig. 7.

Through the above analysis, it can be concluded that the freeze–thaw cycle can change the particle roundness, and repeated freeze–thaw cycles will lead to the increase or decrease of the particle roundness.

In order to describe the influence process of freeze–thaw on particle roundness, the conceptual diagram is expressed as follows: Due to the freeze–thaw effect, the particles are fragmented, the roundness is decreased, and the particle size is decreased. The fragmented particles have edges and small roundness values. Repeated freeze–thaw result in particle edge fragmentation and increased particle roundness.

**Figure 8:**
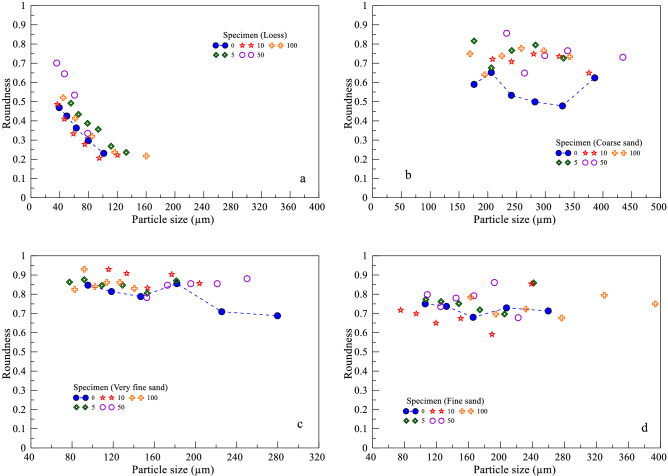
Changes in particle size and roundness of each type of soil after freeze–thaw cycles. (**a** Specimen (L); **b** Specimen (CS); **c** Specimen (VFS); **d** Specimen (FS)).

From the analysis of Fig. 6, it can be concluded that repeated freeze–thaw cycles can lead to an increase or decrease in particle roundness. Figure 7 shows that under repeated freeze–thaw cycles, the particle size also changes while the particle roundness changes. In order to analyze the relationship between particle size and roundness after freeze–thaw cycles, a graph of particle size and roundness changes for each type of soil after freeze–thaw cycles was drawn (Fig. 8).

In Fig. 8, the green dotted line represents the initial roundness distribution of the specimen. It can be seen from the figure that the roundness of loess decreases with the increase of particle size, while the roundness of coarse sand, very fine sand and fine sand does not decrease, but fluctuates within a certain range. The overall roundness of very fine sand is the largest, followed by fine sand and coarse sand and the smallest is loess. With the increase of freeze–thaw cycles, the roundness of different particle sizes increased or decreased, which was due to the particle fragmentation or rounding leading to the change of roundness. In the 10th freeze–thaw cycle, the roundness of loess and fine sand is both less than the initial value, while the roundness after other freeze–thaw cycles is greater than the initial value. This is because the 10th freeze–thaw cycle caused the particles to be fragmented, resulting in a decrease in roundness. However, the roundness of coarse sand and very fine sand varied with the number of freeze–thaw cycles. Overall, the roundness of the four soil specimens increased after 100 freeze–thaw cycles.

**Figure 9:**
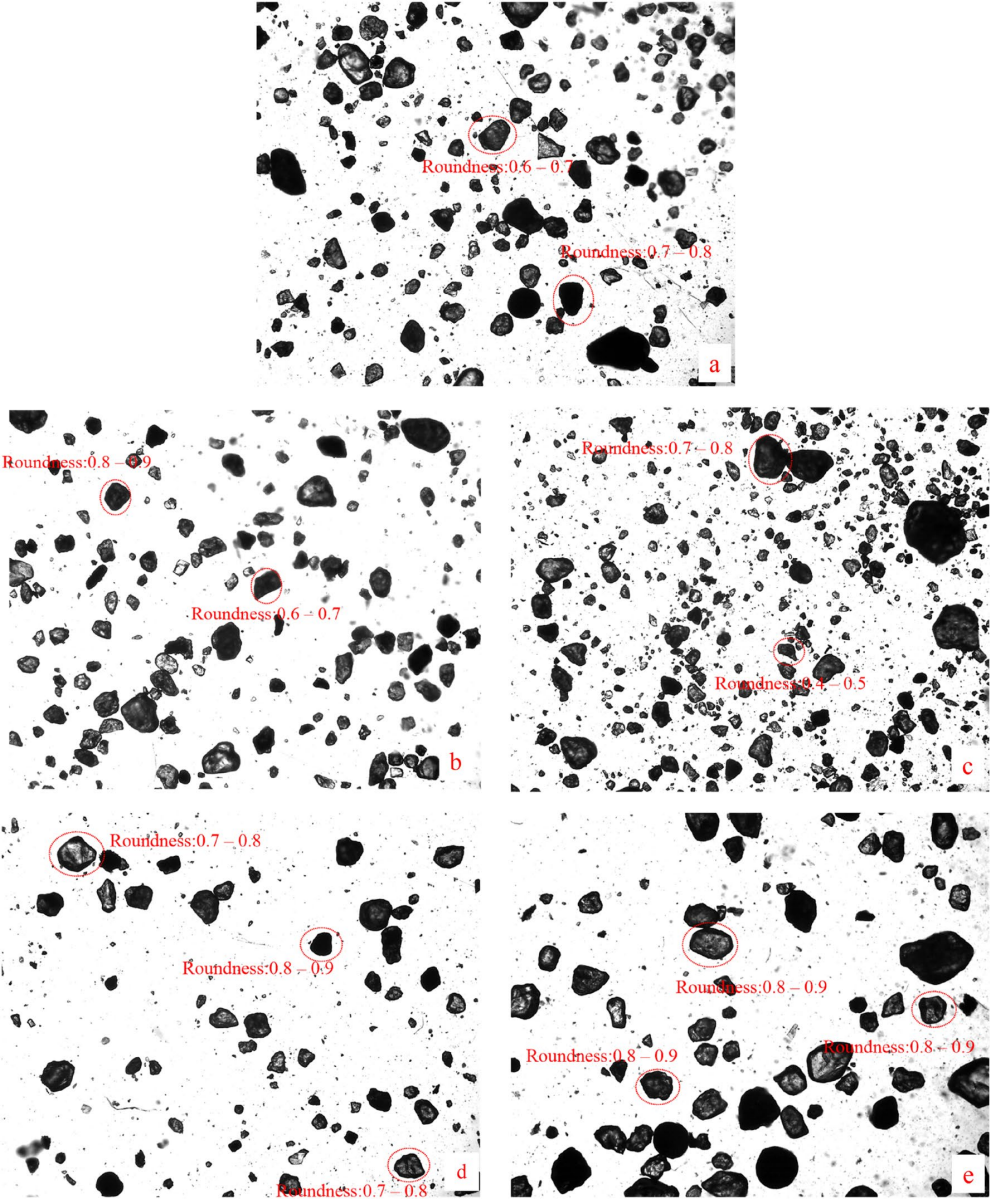
Specimen (FS) particle images after the freeze–thaw cycles (**a** 0 freeze–thaw cycles; **b** 5 freeze–thaw cycles; **c** 10 freeze–thaw cycles; **d** 50 freeze–thaw cycles; **e** 100 freeze–thaw cycles).

Next, take the change of specimen (FS) after different freeze–thaw cycles as an example to analyze the change of its roundness. The images of specimen (FS) after different freeze–thaw cycles were obtained by a particle image processor (a Malvern Panalytical). To facilitate analysis, single particle image is extracted from particle image for analysis. The single particle images of 5,10,50 and 100 freeze–thaw cycles were extracted from the corresponding images after different freeze–thaw cycles. From Fig. 9, it can be seen that the roundness of the particles increased after 5 freeze–thaw cycles. After the 10th freeze–thaw cycle, the particles were fragmented and the roundness of the particles decreased, i.e., the case of freeze–thaw effect leading to the decrease of the roundness of the particles in Fig. 7. After 50 and 100 freeze–thaw cycles, the roundness of the particles increased, i.e., the case in Fig. 7 where the freeze–thaw effect led to an increase in the roundness of the particles. This is consistent with the variation pattern of roundness of the specimen (FS) in Fig. 8.

## Discussion and analysis

The shape and surface texture of the particles are closely related to the geological environment. As described in the introduction to the text, the shape and surface texture of the particles provide vital information regarding the origin, transport and deposition history^5–11^. In aeolian environment, there are three ways soil particles are moved by wind: surface creep, saltation, and suspension^15,27–29^. Due to both size-selective transport and saltation abrasion, the eolian environments contain mostly well-sorted, subrounded grains^15–18^. The roundness of aeolian deposited particles increases with the transport distance from the source area^30^. Similarly, when the transport mode is fluvial transport, the particle size decreases and the roundness and sphericity increase due to the shape sorting and increase of transport distance^31–38^.

**Figure 10:**
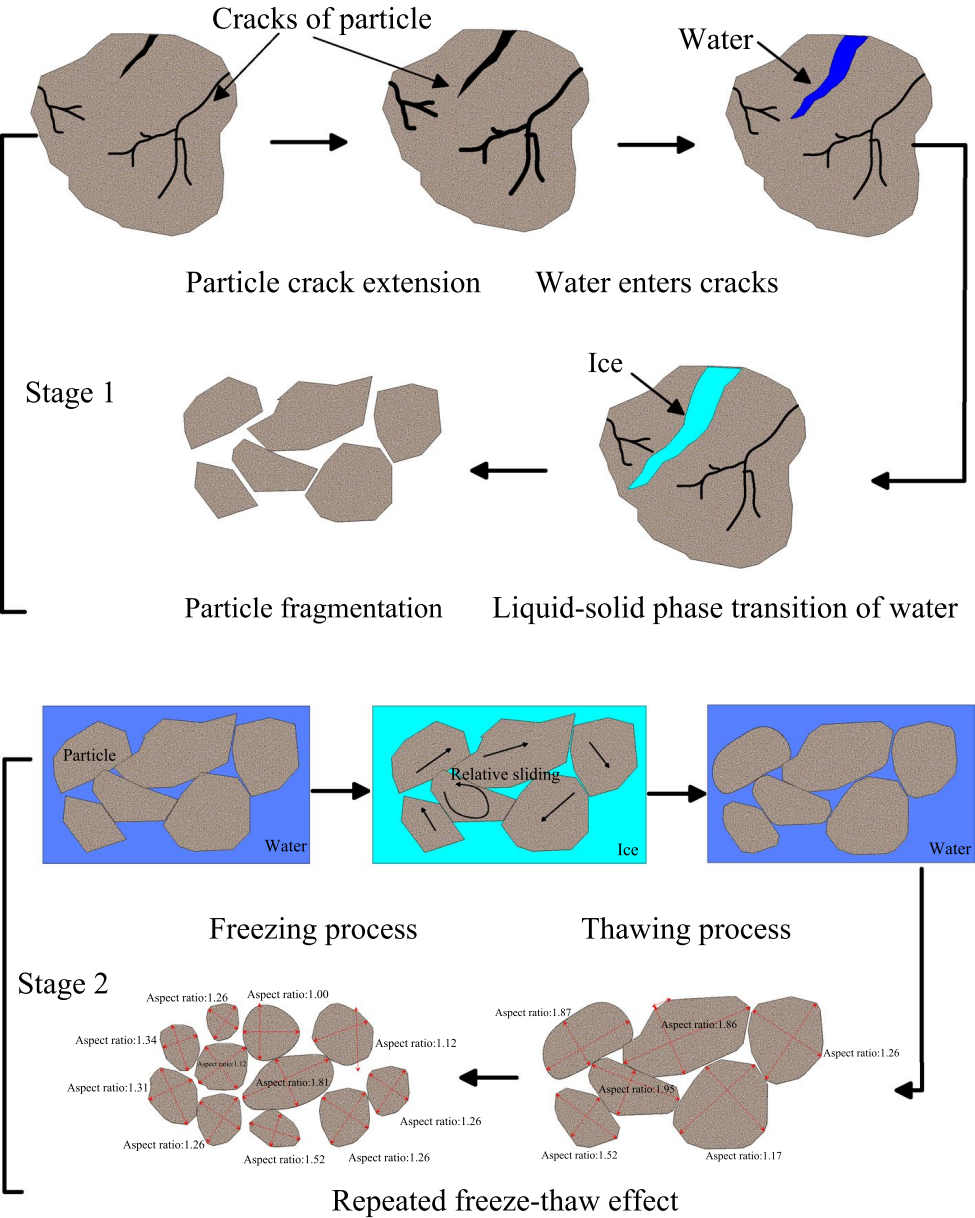
Conceptual diagram of freeze–thaw effect changing particle aspect ratio and roundness (Note that the diagram applies to large particle sizes).

From the above literature, it can be seen that both fluvial and wind transportation can lead to the increase of roundness of the particles. However, the freeze–thaw effect causes changes in particle morphology that are different from aeolian and river transport processes, and also have their own characteristics. The process of particle size, aspect ratio and roundness change caused by freeze–thaw effect is shown by concept diagram. As shown in Fig. 10. There are two main stages in the freeze–thaw effect leads to particle size, aspect ratio and roundness change. In the first stage, driven by the temperature tension and the water phase change in the soil, the cracks will occur on the surface of coarse particles. When water enters the cracks, the water phase becomes ice (volume increases by 9%), which leads to the expansion and penetration of particle cracks, and ultimately leads to particle fragmentation, particle size decreases, aspect ratio and roundness changes. In the second stage, under the freeze–thaw effect, the relative sliding between particles occurs, the edge of particles is fragmented, and the roundness of particles increases. Under repeated freeze–thaw action, the aspect ratio of particles decreases, and the percentage of particles with aspect ratio of 1.26 is the majority.

Through the roundness analysis of four specimens, it is found that after 100 freeze–thaw cycles, the particle’s roundness increases. That is, the cryogenic weathering process will lead to the increase of particle roundness. This is consistent with that when the transport is the fluvial medium, the roundness of particles increases after a distance of flow transport^12^. It is also consistent with the fact that the main morphological feature of particles is well-sorted, subrounded grains in aeolian environment^15–18^. Therefore, the increase of particle roundness after 100 freeze–thaw cycles cannot be used as a basis for judging whether the particles have undergone cryogenic weathering.

However, after repeated freeze–thaw effect, in addition to the roundness of particles increased, the aspect ratio of particles also decreased. After freeze–thaw, the particle with aspect ratio of 1.26 has the highest percentage content. As shown in Fig. 10. However, whether the aspect ratio of 1.26 is a unique feature of cryogenic weathering and whether it can be used to determine the particles have undergone cryogenic weathering need further research and verification.

## Conclusion

The particle shape and surface structure provide important information about the origin, transport and deposition history. This study was conducted to find the unique morphological characteristics of the particles under the influence of cryogenic weathering. It is hoped that this study helps to understand the geometric characteristics of soil primary mineral particles under the action of cryogenic environments, and also helps to discern whether the particles have experienced the action of cryogenic environments, which is important for the study of cryogenic soil in cold environments. By analyzing the aspect ratio and roundness of loess (L), coarse sand (CS), very fine sand (VFS) and fine sand (FS) after freeze–thaw cycles, it is found that:

Through the analysis of the aspect ratio and roundness of four soil specimens after different freeze–thaw cycles, it is concluded that freeze–thaw effect will change the roundness and aspect ratio of particles, and the aspect ratio of particles is mainly distributed between 1 and 4, accounting for more than 98% of the percentage of particle aspect ratio distribution. In addition, it is found that when the aspect ratio of particles is 1.26, the percentage content of particles is the largest and the particles are the most stable. The proportion of particles with aspect ratio greater than 4 is small and the particles are easy to break.

Repeated freezing and thawing can not only lead to fragmentation particle edges and increased particle roundness, but also to fragmentation large-size particles and reduced particle roundness. Compared with the roundness before freeze–thaw and after 100 freeze–thaw cycles, the roundness of coarse sand increased by 0.1715. The roundness of coarse sand increased the most, which indicates that the roundness of particles with large particle size increased the most.

## Data Availability

Some or all data generated or used during the study are available from the corresponding author by request.
